# The Role of 5-Lipoxygenase and Leukotrienes in Shock and Ischemia-Reperfusion Injury

**DOI:** 10.1100/tsw.2007.34

**Published:** 2007-01-22

**Authors:** Antonietta Rossi, Carlo Pergola, Salvatore Cuzzocrea, Lidia Sautebin

**Affiliations:** ^1^ Department of Experimental Pharmacology, University of Naples Federico II, Naples, Italy; ^2^ IRCCS Centro Neurolesi “Bonino-Pulejo”, Messina, Italy; ^3^ Department of Clinical and Experimental Medicine and Pharmacology, University of Messina, Messina, Italy

**Keywords:** 5-lipoxygenase (5-LO), leukotrienes (LTs), septic shock, nonseptic shock, multiple organ dysfunction syndrome (MODS), intestinal ischemia-reperfusion, cerebral ischemiareperfusion, renal ischemia-reperfusion, myocardial ischemia-reperfusion, hepatic ischemiareperfusion, pulmonary ischemia-reperfusion

## Abstract

The leukotrienes (LTs) are metabolic products of arachidonic acid via the 5-lipoxygenase (5-LO) pathway. The biological activities of LTs suggest that they are mediators of acute inflammatory and immediate hypersensitivity responses. In particular, the 5-LO activation has been proposed to be an important regulator for pathogenesis in multicellular organisms. The role of LTs in tissue damage, associated with septic and nonseptic shock and ischemia-reperfusion, has been extensively studied by the use of 5-LO inhibitors, receptor antagonists, and mice with a targeted disruption of the 5-LO gene (5-LOKO). In particular, several data indicate that LTs regulate neutrophil trafficking in damaged tissue in shock and ischemia-reperfusion, mainly through the modulation of adhesion molecule expression. This concept may provide new insights into the interpretation of the protective effect of 5-LO inhibition, which may be useful in the therapy of pathological conditions associated with septic and nonseptic shock and ischemia-reperfusion injury.

## 5-LIPOXYGENASE AND LEUKOTRIENES

5-Lipoxygenase (5-LO), whose activity was first described in 1976 by Borgeat et al.[1], is the key enzyme in leukotriene (LT) biosynthesis from arachidonic acid (AA).

The purified enzyme is a monomer with an estimated molecular weight between 72 and 80 kDa. The 5-LO gene is >82 kb in length and encompasses 14 exons separated by 13 introns[2]. The 5-LO promoter contains consensus regions for a number of transcription regulators belonging to the Egr, Sp, NF-kB, GATA, myb, and AP families[3]. For full activity, 5-LO requires cofactors, such as calcium and ATP, and interaction with other proteins, such as 5-LO–activating protein (FLAP), which facilitates the docking of AA to 5-LO, and the coactosin-like protein (CLP), which stimulates 5-LO activity and colocalizes with the enzyme[4].

Moreover, the enzyme contains, in the active site, a nonheme iron that is essential for its enzymatic activity[5].Post-translational modifications, i.e., phosphorylation by p38 kinase–dependent mitogen-activated protein kinase (MAPK)[6], phosphorylation on Ser 663 by extracellular signal-regulated kinase (ERK)[7], and the subsequent translocation to the nuclear envelope are also fundamental for the activity of the enzyme[8].

In resting cells, 5-LO occurs as a soluble enzyme either in the cytosol or in the nucleus, depending on the cell type[7]. In neutrophils, cytoplasmic 5-LO associates with the endoplasmatic reticulum and the outer nuclear membrane, whereas in dendritic cells or in alveolar macrophages, it has an intranuclear localization that seems to be correlated with a higher capacity for LT generation[9]. Cell stimulation by various agonists causes 5-LO translocation from soluble compartments to the nuclear membrane and, thus, LT generation. 5-LO comigrates with cytosolic phospholypase A2 (cPLA2) to the nucleus where cPLA2 liberates AA from phospholipids, which is then transferred by FLAP to 5-LO.

The production of LTs begins with the insertion by 5-LO of molecular O2 at carbon-5 of AA to produce 5-hydroperoxyeicosatetraenoic acid (5-HPETE). 5-HPETE can be reduced to 5-hydroxyeicosatetraenoic acid (5-HETE), which can be in turn dehydrogenated to 5-oxo-ETE. More typically, 5-LO catalyzes a second enzymatic step, the conversion of 5-HPETE to leukotriene A4 (LTA4), an unstable intermediate[10] that can be catalytically converted to leukotriene B4 (LTB4) by LTA4 hydrolase[11], or can be conjugated with glutathione by leukotriene C4 (LTC4) synthase (LTC4S), a membrane bound enzyme, to produce the cysteinyl LT (cys-LT), LTC4. The LTC4 is subjected to extracellular cleavage of the glutamic acid (through a $\gamma$-glutamyl transpeptidase) and subsequently the glycine moiety (through a dipeptidase) to provid,e respectively, leukotriene D4 (LTD4) and E4 (LTE4)[12].

LTs are proinflammatory mediators and, in fact, they are predominantly synthesized by inflammatory cells like polymorphonuclear leukocytes (PMNs), monocytes, macrophages, and mast cells. The overproduction of LTs has been associated with allergic diseases[13,14,15], pulmonary fibrosis[16], atherosclerosis[17,18], hyperlipidemia-dependent inflammation of the arterial wall[19], pulmonary hypertension[20], and arthritis[21]. In addition, increased 5-LO expression, and presumably increased LT synthesis, has been associated with several tumor types, such as lung[22], pancreatic[23], bladder[24], breast[25], colon[26], multiforme glioblastoma[27], prostate[28], testicular[29], and esophageal cancer[30]. The role of 5-LO and LTs in carcinogenesis seems to be related to their action on fundamental cellular processes, such as differentiation and proliferation, through the transcription of various cytokines and growth factors[31].

Among LTs, LTB4 is a potent proinflammatory mediator that appears to play a role in various diseases associated with neutrophil infiltration, such as arthritis and shock, due to its chemoattractant properties. Moreover LTB4 and cys-LTs increase the adhesion of leukocytes to endothelial cells, an important step in neutrophil migration, through specific integrins. LTB4 also plays a beneficial role in host defense against bacterial infection, and it has been shown to promote bacterial phagocytosis and killing[32]. It acts principally through the BLT1 receptor[33], which is highly selective for LTB4, and displays only weak affinity for closely related compounds, such as 6-trans-LTB4 and 12-epi-6-trans-LTB4. The BLT1 receptor is necessary to elicit the physiological effects of LTB4 (e.g., recruitment of leukocytes and their adhesion to endothelium and calcium mobilization)[34]. There is also a second LTB4 receptor, the BLT2, which is somewhat less selective for this substance and whose precise function is not currently well understood[35]. Cys-LTs, which are released by leukocytes in response to inflammatory and immunological stimuli, cause contraction of endothelial cells, resulting in an increased permeability of postcapillary venules. The cys-LTs are potent bronchoconstrictors and induce a variety of responses associated with asthma including mucus secretion and airway smooth cell muscle proliferation[36]. LTD4 acts through the cys-LT1 receptor that is highly selective for LTD4 over LTC4, LTE4, and other eicosanoids[37]. LTD4 also interacts with a second receptor, the cys-LT2 receptor, which is less selective and has equal affinity for LTC4[38]. The existence of another receptor (cys-LT3) has been postulated[39]. The cys-LT1 receptor mediates most of the actions of LTD4 on the lung and various selective antagonists including montelukast (Singulair), zafirlukast (Accolate), and pranlukast (Ultair) are available for use in the treatment of asthma[40].

Other 5-LO products, such as 5(S)-HETE and 5-oxo-ETE, seem to have important biological activity. Both these metabolites activate neutrophils and/or monocytes, and recently a $G_{i/o}$-coupled eicosanoid receptor, recognized by 5-oxo-HETE, has been identified[41].

By the use of mice with a targeted disruption of the 5-LO gene (5-LOKO), we have recently demonstrated that this enzyme and its metabolites play a pivotal role in several pathological conditions, such as pleurisy[42], acute pancreatitis[43], colitis[44,45], spinal cord injury[46], septic and nonseptic shock[47,48], and ischemia-reperfusion injury[49,50] characterized by an excessive neutrophil activation by promoting neutrophil migration through an up-regulation of adhesion molecule expression.

In the light of what was reported, the aim of this review is to analyze the results reported in the literature on the role of 5-LO and its metabolites, especially LTs, in some pathological conditions, such as shock and ischemia-reperfusion injury, characterized by a severe neutrophil infiltration that gives rise to tissue injury. In fact, the comprehension of the mechanisms underlying this process, and the role of the enzyme and/or its metabolites, might open new perspectives in the therapy of the organ dysfunction and/or injury associated with these pathological conditions.

## 5-LIPOXYGENASE AND LEUKOTRIENES IN SEPTIC SHOCK

In recent years, the problem of infections has increased significantly, especially in intensive care hospital wards, such as intensive care units, emergency medicine, surgery, and critically ill patient care departments[51].

When local innate immunity is overwhelmed and microbial infection disseminates via the bloodstream, the sepsis syndrome results, characterized by hypotension, hypothermia, poor tissue perfusion, and multiorgan dysfunction. Several factors, such as immunosuppression and antibiotic resistance, have been implicated in the increase of sepsis. Despite the availability of an increasing array of potent antibiotics and intensive medical care, mortality due to sepsis remains high[52,53,54]. A myriad of investigational therapies has had little impact on outcomes of sepsis and novel approaches are required.

Sepsis is a complex syndrome, defined as the presence, or presumed presence, of an infection accompanied by evidence of a systemic response called the systemic inflammatory response syndrome (SIRS)[55], which can develop into conditions of different severity ultimately represented by severe sepsis or septic shock[51]. Severe sepsis is defined as the presence of sepsis and one or more organ dysfunctions (acute lung injury; coagulation abnormalities; thrombocytopenia; altered mental status; renal, liver, or cardiac failure; hypoperfusion with lactic acidosis)[56,57], while septic shock is defined as the presence of sepsis and arterial hypotension (systolic blood pressure <90 mmHg, mean arterial pressure <70 mmHg, or systolic blood pressure decrease >40 mmHg)[55].

Half of all cases of septic shock are caused by gram-negative microorganisms and half of these are associated with a positive blood culture[58]. Moreover, the earliest event in the inflammatory cascade seems to be the release of either a heat-stable endotoxin[59,60,61], which is part of the lipopolysaccharide (LPS) of the gram-negative membrane, or soluble peptidoglycans and lipoteichoic acid from gram-positive membranes[62,63,64]. These molecules use similar transduction pathways to induce macrophage activation and proinflammatory response[65]. LPS binds to LPS-binding protein produced by the liver and facilitates LPS binding to the macrophage receptor CD14. The activation of this receptor through a toll-like molecule (TLR4)[66] is responsible for initiating the transmembrane signaling. The initial responses to endotoxemia are detectable in the microcirculation as a microvascular inflammatory response characterized by activation of the endothelium stimulating these cells from their normal anticoagulant state to a procoagulant state with increased adhesiveness for leukocytes and platelets. The infiltration and accumulation of PMNs represent a crucial event for the development of secondary organ and tissue damage[67,68,69,70]. Moreover, leukocyte/endothelial cell interaction is also induced by the generation of proinflammatory mediators, such as LTs, which up-regulate adhesion molecule expression[71,72,73], as discussed below.

The importance of 5-LO in the induction and development of shock has been established in *in vitro* and *in vivo* studies by the use of chemical inhibitors of 5-LO and genetically engineered mutant mice devoid of the enzyme[47,74]. It is currently accepted that the action of 5-LO is central to immune response, as it is responsible for the generation of LTs, which are secreted and work in an autocrine or paracrine fashion to drive cellular and tissue components of innate immunity [75]. It has been demonstrated that mice lacking 5-LO cannot produce LTs and have an impaired ability to clear bacterial infection[76]. In fact, in a model of *Klebsiella pneumoniae* pneumonia, 5-LOKO mice exhibit enhanced lethality and reduced pulmonary bacterial clearance in respect to wild-type (WT) mice[75]. Subsequent studies have demonstrated that both classes of LTs are necessary for optimal phagocytosis of IgG-opsonized microbes[75,77,78]. However, the overall results concerning the role of LTs in the immune response demonstrate that they have divergent effects during the induction and evolution of septic shock. Initially, they participate in local innate immune control. However, if the severity of infection overwhelms local immunity and microbial dissemination ensues, cys-LTs (but not LTB4) contribute to the deleterious effects on the vasculature, resulting in vascular leak, hypotension, and inadequate tissue perfusion[79]. In fact, in addition to their actions on airways, cys-LTs have long been recognized to increase microvascular permeability in various organs[80,81,82,83]. It has been reported that intraperitoneal injection of LPS in rats leads to a strong increase of LTC4S messenger RNA (mRNA) levels after approximately 1 h, particularly in the heart, brain, adrenal glands, and liver. After 6 h, LTC4S mRNA returns to basal levels, concomitant with a 4.9-, 4.0-, 2.9-, and 2.3-fold induction of LTC4S protein in brain, heart, liver, and adrenal gland, respectively. Hence, challenge with LPS *in vivo* causes an organ-selective, local priming for LTC4 synthesis[84]. Thus LTs, although initially playing a protective role during septic shock development, have a detrimental role as demonstrated by several experimental studies indicating salutary effects of LT inhibitors and antagonists in endotoxin shock[85]. In fact, it has been demonstrated by the use of 5-LO inhibitor, that LTs cause pulmonary hypertension, systemic hypotension[86], and hypothermia[87], and increase vascular permeability during bacteremia[86]. Moreover, in a model of endotoxic shock, the treatment with BW A137C, a 5-LO inhibitor, attenuated acute microvascular injury produced by LPS. Similarly, the administration of MK-886, LT biosynthesis inhibitor, attenuated the hypotension and partially reversed the impaired vascular responsiveness observed in a rabbit model of endotoxic shock[88].

In addition to LTs, the excessive formation of other inflammatory mediators, such as prostaglandins (PGs) and nitric oxide (NO), has been implicated in the pathogenesis of septic shock. An interesting aspect to be considered is the balance between the protective role of constitutive NO synthase (NOS) and the detrimental actions of 5-LO and its products in the maintenance of microvascular integrity in the early stage of sepsis[89]. This is not surprising since a cross-talk between these mediators has been described in several cell types. For example, a prolonged exposure to LPS inhibits macrophage 5-LO metabolism via induction of NO synthesis. In fact, it has been showen that pretreatment of macrophages with LPS causes time- and dose-dependent induction of NOS and a suppression of LT synthesis. Inhibition of 5-LO by LPS was reproduced by the use of NO donors, and was abrogated by inhibitors of constitutive and inducible NOS[90,91]. Interestingly, the reactive oxygen species and peroxynitrite seem to be involved in this regulation[92]. Moreover, it has been demonstrated that the 5-LO pathway can be also regulated by PGs. In fact, PGE2, via its EP4 receptor, negatively regulates 5-LO translocation and activation through a complex series of events[93]. Recently, we have found that in *in vitro* models of inflammation, such as mouse-peritoneal macrophages activated with LPS and interferon-$\gamma$, the deletion of the gene encoding for 5-LO or the enzyme activity inhibition (zileuton) corresponded to a negative modulation of the cyclooxygenase (COX) pathway. Moreover, we have demonstrated that LTC4 is able to increase PG production by up-regulation of COX-2 expression, an effect mediated by Erk-1/2 activation. The LT-induced increase in PG generation, as well as MAPK activation, was dependent by a specific ligand-receptor interaction[94]. Thus, the mutual interaction between LTs, PGs, and NO may further modulate the development of septic shock.

As reported above, shock can develop into multiple organ dysfunction syndrome (MODS), also known as multiple organ failure (MOF), with the lung as the first organ involved. In particular, LTs have been implicated as possible mediators of endotoxin-induced acute lung injury. In fact, the presence of LTs in the bronchoalveolar lavage fluid of patients with sepsis, as well as increased cys-LT levels in the lung tissue of endotoxin-challenged rodents, has been reported[95,96]. Moreover, infusion of LTs into animals produces acute lung injury resembling the clinical presentation of endotoxemia including pulmonary hypertension and increased vascular permeability, resulting in pulmonary edema and hypoxemia[97,98]. We have demonstrated in collaboration with Thiemermann and coworkers that lung, ileum, liver, renal, and pancreatic dysfunction and injury, caused by endotoxemia, as well as PMN infiltration [myeloperoxidase (MPO) activity] in the lung and ileum, were reduced in rats treated with 5-LO inhibitor zileuton and in 5-LOKO mice. Zileuton also reduced the LPS-induced expression of β2 integrins CD11b/CD18 on rat leukocytes. Thus, zileuton seems to protect organs against endotoxin-induced dysfunction and injury by inhibiting the LT synthesis, thereby reducing the LT-induced stimulation of β2-integrin–dependent adhesion and the subsequent recruitment of neutrophils. We propose that endogenous 5-LO metabolites enhance the degree of MOD and/or injury caused by severe endotoxemia by promoting the expression of these adhesion molecules[47].

Altogether, all the data reported in the literature indicate that 5-LO plays pivotal role in response to endotoxic shock, and that inhibitors of 5-LO might be useful in the therapy of the organ dysfunction and/or injury associated with endotoxemia.

## 5-LIPOXYGENASE AND LEUKOTRIENES IN NONSEPTIC MULTIPLE ORGAN DYSFUNCTION SYNDROME

As reported above, MODS is defined as a cumulative sequence of function-progressive deterioration occurring in several organ systems, frequently seen after shock, multiple trauma, severe burns, or pancreatitis[99,100,101]. MODS remains a principal cause of death after severe shock or trauma, not only in the presence, but also in the absence, of sepsis[102,103,104,105,106,107,108]. Several animal models have been used to understand the pathophysiological mechanisms underlying nonseptic MODS. Among them, administration of zymosan, a nonbacterial and nonendotoxic agent, produces acute peritonitis and MODS in experimental animals, characterized by functional and structural changes in the lung, liver, intestine, and kidneys[109,110,111,112,113,114]. It has been reported that zymosan administration to mice causes signs of both peritonitis and organ injury within 18 h[115,116]. The onset of the inflammatory response is associated with systemic hypotension, maximal cellular infiltration, exudate formation, edema, and release of inflammatory mediators[115,116,117,118,119]. It has been demonstrated that intraperitoneal administration of zymosan to mice resulted in marked biosynthesis of LTC4 and LTB4. In particular, LTC4 increased between 30 and 60 min and sustained for several hours, whereas LTB4 increased in a biphasic manner with a peak between 2 to 3 h[120]. It has also been demonstrated that macrophage-depleted rats are protected from systemic toxicity and mortality after zymosan administration[121,122,123,124], indicating that macrophages play an important role in zymosan-induced inflammatory processes, probably by activating the synthesis of cytokines and proinflammatory mediators such as NO, PGs, and LTs[112,113,114,120,125].

Other results support the pivotal role of LTs in the zymosan-induced peritonitis model. In fact, edema associated with zymosan-induced peritonitis was markedly reduced in animals lacking FLAP[126], and it has been reported that 5-LO inhibitors and LTB4 receptor antagonists are effective in preventing the development of organ failure since they reduce neutrophil infiltration[120]. Moreover, it has been demonstrated, by the use of LTA4 hydrolase-deficient mice, that LTB4 influences the cellular component of zymosan-induced peritonitis[127]. We have demonstrated that the absence of 5-LO (5-LOKO mice) attenuates the development of zymosan-induced peritonitis; the PMN infiltration of the lung and intestine (histology and MPO activity); the degree of liver, kidney, and pancreas organ dysfunction (biochemical markers); and the degree of lung and intestine injury (histology) caused by injection of zymosan[48]. The mechanisms of the anti-inflammatory effect of the absence of functional 5-LO are not entirely clear. Our findings demonstrate that 5-LO exerts a role in zymosan-induced nonseptic shock by the regulation of neutrophil recruitment, both at the rolling and firm adhesion phase. In fact, it appears that the genetic inhibition of 5-LO reduced the expression of adhesion molecules, such as P-selectin and ICAM-1. These endothelial adhesion molecules are major regulators of neutrophil trafficking, regulating the process of neutrophil chemoattraction, adhesion, and transmigration from the vascular bed to the injured tissue[128,129]. Thus, these results support the view that the overproduction of LTs contributes to the development of MODS and confirm the concept that 5-LO

regulates neutrophil trafficking also in conditions associated with nonseptic shock through the positive modulation of adhesion molecule expression.

## 5-LIPOXYGENASE AND LEUKOTRIENES IN ISCHEMIA-REPERFUSION INJURY

### Intestinal Ischemia

Intestinal ischemia is generally the result of arterial occlusion by thrombi or emboli and, more frequently, by nonocclusive processes, such as in situations of low mesenteric flow, which occurs in cardiac insufficiency, sepsis, and administration of alpha-adrenergic agents or digitalics[130,131]. The arteries most compromised by the obstruction are the celiac trunk (or celiac artery), superior mesenteric (or cranial) artery, and inferior mesenteric (or caudal) artery[132].

Ischemia leads to hypoxia, which initiates a series of events primarily related to activation of platelets and release of their vasoconstrictor mediators (e.g., thromboxane A2 and 5-hydroxytryptamine) that further restrict blood flow to the ischemic area. If the ischemia is severe enough, the rate of metabolism is diminished and the generation of high-energy compounds (e.g., ATP) subsequently declines. The reduced-energy metabolism eventually leads to a slow, but significant, degree of tissue injury and necrosis. The degree of tissue injury is further enhanced and accelerated by reperfusion. It is important to realize that reperfusion of an ischemic organ is not only associated with local changes. In fact, in some situations, reperfusion is also associated with systemic changes. For example, in a model of ischemia and reperfusion (I/R) of the intestine, local functional alterations include intestinal hyperpermeability as well as morphological changes, such as necrotic injury of the reperfused tissues[133,134]. On the other hand, systemic alterations characteristic of postreperfusion include a progressive fall in the mean arterial blood pressure, release of proinflammatory mediators from the reperfused tissues into the systemic circulation, and ultimately, a decreased survival[135]. A severe form of circulatory shock produced by I/R of the splanchnic organs is the splanchnic artery occlusion (SAO) shock. This type of shock is characterized by a decrease in systemic blood pressure on release of the splanchnic arteries, which leads to a fatal outcome[133,134]. An important component of SAO shock is the endothelial dysfunction[134,135] originally attributed to oxygen-derived free radicals released from both the reperfused endothelium[136] and the activated adherent PMNs[137]. Ischemia-reperfusion is also a stimulus for leukocyte-endothelial cell interaction and migration into tissues. Moreover I/R induces an increase in intestinal P-selectin expression[138] and tissues subjected to I/R injury synthesize significant quantities of AA metabolites[139]. In particular, it has been demonstrated that intestinal mucosal LTB4 and LTC4 synthesis triples after ischemia and I/R[140], and the LTC4 regulates the splanchnic blood flow during ischemia[141], whereas LTB4 plays a pivotal role in endothelial dysfunction by chemoattraction and activation of neutrophils on the surface of vascular endothelial cells[142]. In a model of canine I/R injury, the inhibition of 5-LO with zileuton significantly improved reperfusion, intestinal blood flow, and $VO_2$, and abolishes the I/R-induced increase in mucosal neutrophil infiltration in normothermic I/R injury[140]. Moreover, it has been demonstrated that the 5-LO pathway also plays a significant role in the pathophysiology of hypothermic intestinal I/R injury[143] and hypotension associated with I/R[144]. We have shown, using 5-LOKO mice, that 5-LO mediates leukocyte-endothelial interactions by regulating the expression of P-selectin, E-selectin, and ICAM-1 during I/R. In fact, in 5-LOKO mice subjected to SAO shock, the up-regulation of P-selectin, E-selectin, and ICAM-1 in the intestine and lung was largely attenuated[49]. In particular, the genetic inhibition of 5-LO abolished the expression of P-selectin and E-selectin, and the up-regulation of ICAM-1, but did not affect the constitutive expression of ICAM-1 on endothelial cells. These results suggest, as demonstrated in septic and nonseptic shock, that the inhibition of 5-LO activity may interfere with the interaction of neutrophils and endothelial cells, both at the early rolling phase mediated by P-selectin and E-selectin, and at the late firm-adhesion phase mediated by ICAM-1. Besides, it is interesting to note that the early I/R-induced increase in intestinal P-selectin expression seems to be mediated by 5-LO–dependent NO-inhibitable mechanism[138]. In our experimental model, the absence of an increased expression of the adhesion molecules, in the intestine as well as in the lung tissue of SAO-shocked 5-LOKO mice, was associated with the reduction of leukocyte infiltration, as assessed by the specific granulocyte enzyme MPO, and with the attenuation of tissue damage as evaluated by histological examination. It is noteworthy, however, that tissue MPO activity was not completely abolished. This result is consistent with previous studies demonstrating that constitutive levels of ICAM-1 seem to be sufficient to support a lower degree of CD11/CD18-dependent transendothelial migration of activated neutrophils[145]. Thus, as previously underlined, the concept that 5-LO regulates neutrophil trafficking through up-regulation of adhesion molecules may provide new insights into the interpretation of reports that demonstrate the protective effect of 5-LO inhibition in experimental models of I/R injury and inflammation.

## Cerebral Ischemia

Transient cerebral ischemia initiates a complex series of metabolic events which ultimately lead to neuronal death. One critical event is the degradation of membrane lipids and subsequent accumulation of free fatty acids, particularly AA[146]. Accumulation of AA is greater in brain regions most susceptible to I/R injury[147] and is a index of ischemic damage[148,149]. Brain damage caused by I/R is due, in part, to secondary injury from inflammation[150,151] and the degree of inflammation is exacerbated by increased lipid peroxidation, which ultimately increases neuronal death[152]. The importance of 5-LO in stroke has been proven by several authors[153,154,155,156] who have demonstrated that 5-LO expression as well as LT levels are elevated in the ischemic brain[157,158,159,160], indicating an important role for 5-LO in cerebral ischemia. In particular, Zhou et al.[154] demonstrated that the expression of 5-LO, both mRNA and protein, increases in the ischemic core 12 to 24 h after reperfusion, whereas in the boundary zone adjacent to the ischemic core, the increase was observed after 7 to 14 days. The increased 5-LO expression was primarily localized in the ischemic core at 24 h; in the neurons and in the proliferated astrocytes, in the boundary zone, 14 days after reperfusion. Moreover, the cys-LT levels in the ischemic brain were increased 3 to 24 h after reperfusion, declined to the control level after 3 days, and moderately increased again after 7 days, whereas the levels of LTB4 were increased mildly 3 h after reperfusion, but substantially after 7 to 14 days, at 3 h and 7 days after reperfusion, respectively. The first peak (3 h) was earlier than that of 5-LO mRNA or protein expression (24 h) and the possible explanation for the difference is that 5-LO might be activated before its mRNA or protein expression[154]. Recently, it has been shown that the mRNA expression of cys-LT receptor-1 is increased in the brain after cerebral ischemia, in rats, and that cys-LT receptor 1 antagonists protect against cerebral ischemic injury[161]. In a gerbil model of transient forebrain, it has been reported that, during reperfusion, neurons exhibit dense 5-LO immunoreactivity and the enzyme is redistributed from cytosol to particulate fraction after 3-min reperfusion. The LTC4 levels were increased in all forebrain regions during reperfusion and postischemic increases were inhomogeneous; a greater increase was observed in the hippocampus than in cerebral cortex. Thus, Ohtsuki et al. suggested that reperfusion, which was associated with translocation of cytosolic 5-LO to membranes, induced the biosynthesis of LTC4 that may mediate irreversible reperfusion injury in the hippocampal neurons[157].

Recently, 5-LO has been shown to be involved in ischemic-like injury in neuronal PC12 cells[162]. However, in an animal model of focal cerebral ischemia, no difference in cerebral infarct size has been found between 5-LOKO and wild-type mice[163].

The overall data herein reported suggested that the involvement of 5-LO in cerebral ischemia needs to be further investigated.

## Renal Ischemia

The temporary discontinuation of renal blood supply is encountered in many clinical situations, such as kidney transplantation[164], partial nephrectomy[165], renal artery angioplasty[166], cardiopulmonary bypass[167], aortic bypass surgery[168], accidental or iatrogenic trauma[169], sepsis[170], hydronephrosis[171], and elective urological operations[172]. Thus, renal ischemia is a major cause of acute renal failure (ARF), which remains, despite significant advances in critical care medicine, a major clinical problem, producing grave morbidity and mortality that has not significantly decreased over the last 50 years[173]. As observed in other ischemic organs, also when the return of blood flow to ischemic tissue results in the recovery of normal functions, the tissue may be paradoxically injured during the process of reperfusion[174]. I/R injury of the kidney is characterized by a series of events including changes in vascular tone, enhanced vascular permeability to plasma proteins, structural alterations in renal tubule, and accumulation of activated neutrophils[175] through the collective action of chemotactic mediators[176], such as chemokines and 5-LO metabolites[177,178]. Among 5-LO products, LTB4 seems to be a key mediator in the pathophysiology of the renal dysfunction caused by I/R of the kidney[179]. In fact, it has been demonstrated that the cys-LT1 receptor antagonist, montelukast, reverses I/R-induced oxidant responses and improves microscopic damage and renal function. It seems likely that montelukast protects kidney tissue by inhibiting neutrophil infiltration, balancing oxidant-antioxidant status, and regulating the generation of inflammatory mediators[180]. In collaboration with Thiemermann and coworkers, we have demonstrated for the first time that the inhibition of 5-LO activity with zileuton reduced the renal dysfunction and injury caused by bilateral occlusion and reperfusion of mouse kidneys[50]. Bilateral renal I/R in the mouse resulted in a significant increase in plasma levels of LTB4 that was almost abolished by treatment of wild-type mice with zileuton. To confirm that the reported beneficial effects of zileuton were indeed caused by inhibition of 5-LO (rather than a nonspecific effect), we have subsequently compared the effects of bilateral renal artery occlusion and reperfusion in 5-LOKO with those obtained using their wild-type littermates. The degree of renal dysfunction, injury, and inflammation were reduced in 5-LOKO mice indicating, as observed with the inhibition of the enzyme activity, that 5-LO and its metabolites contribute to the pathophysiology of renal I/R injury. Moreover, we have demonstrated that zileuton reduced the expression of ICAM-1 caused by I/R of the kidney in wild-type mice, with a similar reduction in ICAM-1 expression in 5-LOKO mice. The accumulation of PMNs was also reduced in kidneys from 5-LOKO mice or in kidneys from wild-type mice treated with zileuton, which had been subjected to I/R. Thus, inhibition of 5-LO activity reduces PMN accumulation after I/R of the kidney. This is not entirely surprising given that the 5-LO metabolite, LTB4, is a potent chemokine. It should be noted that the renal dysfunction and injury observed in 5-LOKO or wild-type mice treated with zileuton was not entirely abolished. In addition, the degree of inhibition of plasma urea and creatinine were not as complete as that of plasma aspartate aminotransferase. This could be due to many other pathophysiological mechanisms, independent of LTs and/or related to an enhanced inflammatory response, contributing to the observed injury during ischemia and/or reperfusion. These mechanisms may include (but are not limited to) the generation of reactive oxygen and nitrogen species[181], an enhanced formation of NO[182], modification of endogenous lipoxin generation[183], or the activation of the nuclear enzyme poly(ADP-ribose) polymerase[184].

The role of 5-LO metabolites in renal I/R is supported by clinical data demonstrating that zileuton reduces the formation of LTB4 in rectal dialysates as well as the associated inflammatory response in patients with inflammatory bowel syndrome[185].

## Myocardial Ischemia

Ischemic heart disease is the leading cause of mortality in industrialized countries. Myocardial ischemia is most often due to atherosclerotic plaques that reduce the blood supply to a portion of myocardium. Initially, the plaques allow sufficient blood flow to match myocardial demand, however, when myocardial demand increases, the areas of narrowing may become clinically significant and precipitate angina. In particular, the acute coronary syndrome occurs when an unstable plaque ruptures and activates coagulation at the site, blocking blood flow and causing ischemic injury to the heart.

Most of the treatments available for ischemic insults including myocardial infarction and angina are directed toward preventing the tissue damage inflicted at the time of reperfusion when the coronary flow is restored by releasing the occlusion. As reported for other organs, ischemia, with subsequent reperfusion of the myocardium, is associated with inflammation, and it is well known that inflammation and proinflammatory mediators, such as AA metabolites, play a role in atherogenesis, atherosclerotic plaque progression, and acute coronary syndrome[186]. In fact, experimental cardiac ischemia results, in some animal models, in the activation of 5-LO and of proinflammatory LT production by the affected myocardium. It has been demonstrated that LTs are released during episodes of myocardial ischemia, supplying clinical evidence for involvement of their biosynthetic enzyme, 5-LO, during and after acute myocardial infarction and unstable angina attacks. In fact, the urinary excretion of LTE4, the major urinary metabolite of cys-LTs in humans[187], as well as LT levels in systemic artery blood, are considerably higher in patients with diagnosis of acute myocardial infarction (AMI). In particular, these levels are elevated during the acute stage of AMI and decreased to near-normal control levels by 1 month after the attack[188]. Thus, since LTC4 levels are increased in acute cardiac syndrome, it has been suggested that the measurement of LTC4 levels might be useful in clinical diagnosis and management of acute coronary syndromes[189]. Besides, it has been suggested that LTC4 is involved more in prolonged than in transient myocardial ischemia[190], and that LTD4 has a negative inotropic and chronotropic effect in isolated rat hearts with chronic myocardial infarction[191]. Intravenous treatment with BAY X1005, LT synthesis inhibitor, reduced the mortality rate, protected against the marked electrocardiogram derangement, and abolished the significant increase in plasma creatinine phosphokinase activity and cardiac tissue MPO activity induced by coronary artery ligation in rabbit[192]. Moreover, as reported for other I/R models, LTB4 regulates neutrophil infiltration associated with myocardial infarction[193]. Evidence for the involvement of LTs in acute coronary syndrome derive also from their profound effects on cardiac function, which may be mediated through effects on both coronary blood flow and cardiac contractility. In fact, LTs are also capable of altering blood flow to several vascular beds and, when synthesized endogenously and released, may play an important role in the regulation of the peripheral circulation. In addition to their effects on vascular smooth muscle and myocardium, the LTs increase the permeability of blood vessels[194]. No more recent data are available concerning the role of 5-LO on myocardial I/R injury.

However, over the last few years, the attention on myocardial ischemic disease has been addressed to polyunsaturated fatty acids. It has been demonstrated that long-chain polyunsaturated fatty acids, such as eicosapentaenoic acid and AA, protect the heart against I/R-induced injury[195]. In particular, it has been recently recognized that another less-well-characterized metabolic pathway of AA, the cytochrome P450 (CYP450) pathway, may have important cardiovascular effects. Several lines of data support the possibility that certain CYP metabolites resulting from the hydroxylation of AA, such as 20-HETE, are potent vasoconstrictors and may produce detrimental effects in the heart during ischemia and proinflammatory effects during reperfusion. On the other hand, a group of regioisomers resulting from the epoxidation of AA including 5,6-, 8,9-, 11,12-, and 14,15-epoxyeicosatrienoic acid (EETs) have been shown to reduce ischemic and/or reperfusion injury in the heart and vasculature[196]. Thus, at the present time, research is mainly focused on these AA metabolites rather than LTs or other 5-LO metabolites.

## Hepatic and Pulmonary Ischemia

Few results concerning the role of 5-LO in hepatic and pulmonary ischemia are present in the literature and are mainly related to their level measurement.

A common consequence of major liver surgery and liver transplantation is I/R injury. In liver surgery, I/R injury (warm injury) follows hepatic inflow occlusion used to reduce blood loss. In a liver graft, I/R injury (after a combination of cold and warm ischemia) occurs after reperfusion of the graft, although the mechanisms responsible for liver I/R injury following cold and warm ischemia are somewhat different. However, the major damage observed in both warm and cold ischemic livers is caused by the reperfusion step when O2 and blood nutrients interact with hepatocytes and other cell types present in the liver. This leads to injury and, ultimately, the death of hepatocytes and loss of liver function. The hepatic I/R induces local (liver) as well as remote (lung) organ injuries, which are characterized, as reported for other models of I/R, by the accumulation of activated neutrophils and tissue edema resulting from increased vascular permeability during the reperfusion period[197,198]. It has been reported that LTs are associated with several liver injuries, such as fulminant hepatitis[199], liver cirrhosis[200], and hepatic I/R injury[201]. Moreover, it has been demonstrated that cys-LT production in the liver is increased following hepatic I/R associated with the development of hepatic edema and dysfunction. In fact, in the hepatic tissue, after 12 and 24 h reperfusion, the cys-LT, but not LTB4, levels are increased four- to five fold compared to controls, and this is accompanied by the enhancement of hepatic edema and plasma alanine transferase (ALT) elevation. Thus, only cys-LTs seem to contribute to the inflammatory process associated with this condition and to exert hepatotoxicity[202]. However, in contrast, Matsui et al.[203] have demonstrated that the content of LTB4 in the liver increases during ischemia similarly to ALT levels and hepatic MPO activity. Therefore, the involvement of LTs needs further investigations.

Lung transplantation is a widely accepted treatment of choice in patients with various end-stage pulmonary diseases. Recent progress in graft methods, surgical techniques, and postoperative management has improved the clinical outcome of such transplants. However, I/R lung injury occurs in up to 22% of patients after lung transplantation and is still the main cause of death during the first month after surgery[204]. It has been demonstrated that lower torso ischemia followed by reperfusion leads to respiratory failure associated with sequestration of neutrophils in the lung[205,206]. The accumulation and activation of these inflammatory cells is believed to be caused by chemotactic agents, such as LTB4, generated by the ischemic tissue and released into the circulation[207]. It has been demonstrated that overexpression of LTB4 receptor in leukocytes of transgenic mice dramatically increased PMN trafficking to skin microabscesses and lungs after I/R, whereas mice deficient in 5-LO showed diminished PMN accumulation in reperfused lungs[208]. Moreover, the role of LT in I/R injury is suggested by reports showing that after limb ischemia-induced lung injury[209], there is an increase in LTB4 plasma levels as well as in blood and bronchoalveolar lavage fluid LTC4 level[210]. Moreover, in the same model, it has been demonstrated that LTs mediate neutrophil sequestration and lung edema after hind-limb ischemia[207]. However, as mentioned for hepatic I/R, the role of 5-LO and LTs in I/R lung injury has not been completely understood.

## CONCLUSIONS

The overall results reported in the literature support the view that 5-LO and its metabolites, particularly LTs, contribute to multiple organ injury and dysfunction, as well as systemic inflammatory response syndrome and MODS associated with septic and nonseptic shock. Both shock and I/R are characterized by a severe neutrophil infiltration and increased vascular permeability that give rise to tissue injury. The general role of 5-LO seems to be related mainly to the control of vascular permeability and, through an up-regulation of adhesion molecule expression, neutrophil trafficking (Fig. 1). However, conflicting opinions on the actual role of this AA pathway are present in the literature. Thus, the comprehension of the mechanism underlying the role of the enzyme and the relative contribute of its metabolites will improve the knowledge of the

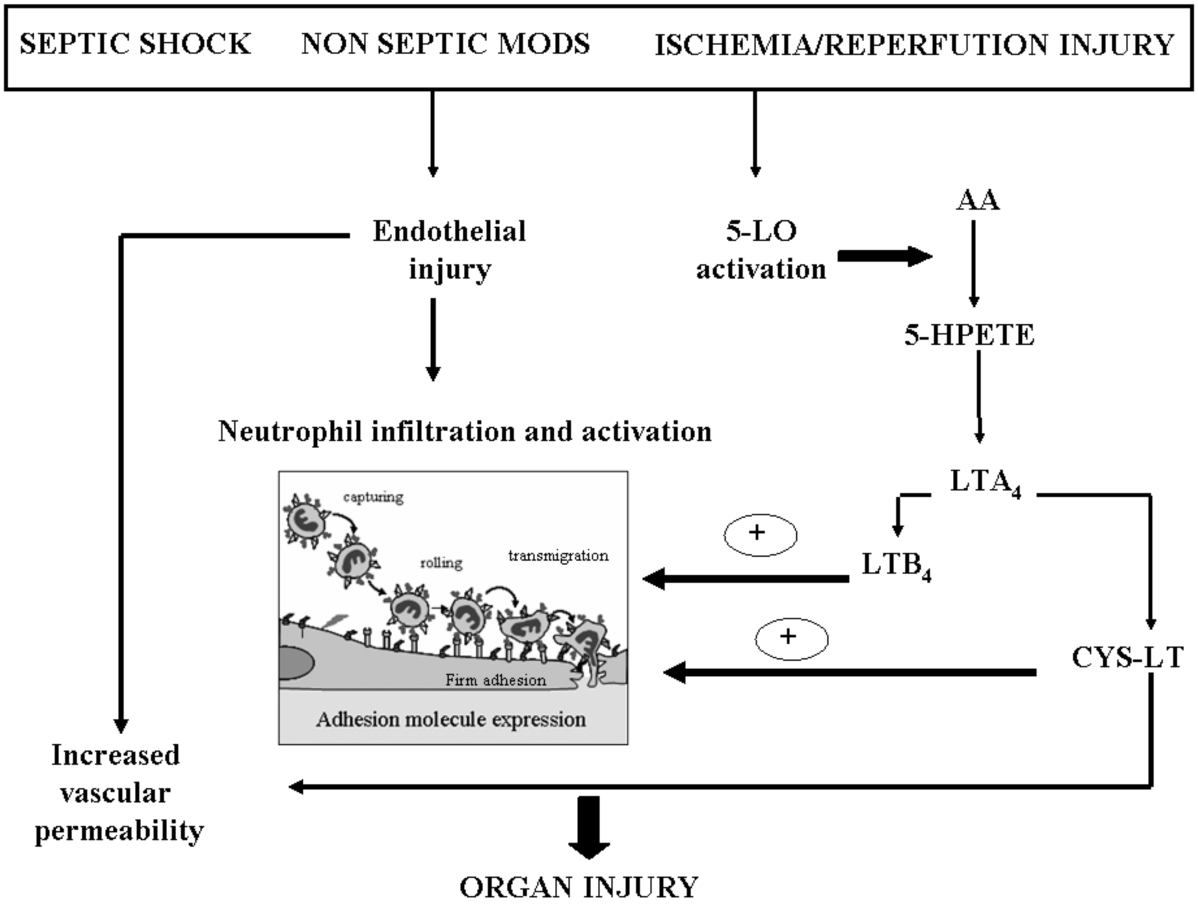

**FIGURE 1.** Proposed scheme for the role of 5-LO in septic shock, nonseptic MODS, and I/R injury.

etiopathogenesis of both shock and I/R, and consequently will open new perspective for the appropriate therapeutic intervention.
